# Recurrent Cellulitis Revealing *Helicobacter cinaedi* in Patient on Ibrutinib Therapy, France

**DOI:** 10.3201/eid2903.221329

**Published:** 2023-03

**Authors:** Anne-Laure Roupie, Emmanuel Lafont, Sylvie Fraitag, Agnès Ferroni, Hervé Lécuyer, Olivia Boccara, Emilie Bessède, Philippe Lehours, François Lefrère, Olivier Lortholary

**Affiliations:** University Hospital Necker for Sick Children, Assistance Publique-Hôpitaux de Paris, Paris, France (A.-L. Roupie, E. Lafont, S. Fraitag, A. Ferroni, H. Lécuyer, O. Boccara, F. Lefrère, O. Lortholary);; Paris-Cité University, Paris (A.-L. Roupie, E. Lafont, S. Fraitag, A. Ferroni, H. Lécuyer, O. Boccara, F. Lefrère, O. Lortholary);; French National Reference Center for Campylobacters & Helicobacters, Bordeaux, France (E. Bessède, P. Lehours);; Bordeaux Institute of Oncology, INSERM UMR1312, Bordeaux (E. Bessède, P. Lehours)

**Keywords:** *Helicobacter cinaedi*, cellulitis, ibrutinib, bacteremia, bacteria, France

## Abstract

*Helicobacter cinaedi* bacteremia caused recurring multifocal cellulitis in a patient in France who had chronic lymphocytic leukemia treated with ibrutinib. Diagnosis required extended blood culture incubation and sequencing of the entire 16S ribosomal RNA gene from single bacterial colonies. Clinicians should consider *H. cinaedi* infection in cases of recurrent cellulitis.

A 61-year-old man who had been treated for 4 years with the Bruton’s tyrosine-kinase (BTK) inhibitor ibrutinib as a first-line therapy for chronic lymphocytic leukemia (CLL) sought treatment for a painful rash. He reported 6 previous episodes in the previous 5 months and had been treated with short courses of amoxicillin, amoxicillin/clavulanate, or pristinamycin. The patient denied fever. We noted, on physical examination, 3 painful, infiltrated erythematous lesions with sharp borders: 1 on his left thigh, 1 on his left tibia, and 1 on the right side of his abdomen (Figure 1).

**Figure 1:**
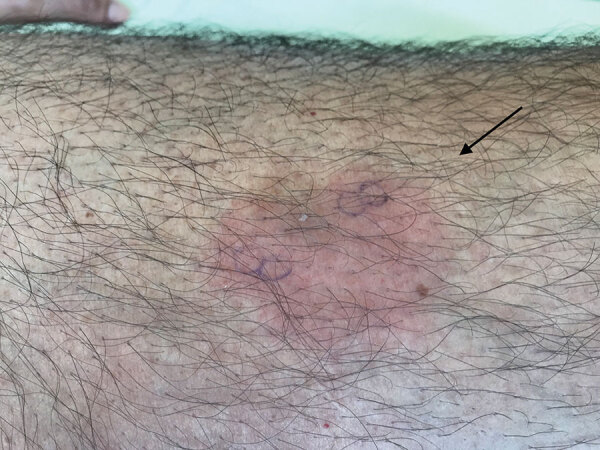
Recurrent skin manifestations revealing *Helicobacter cinaedi* bacteremia in a man on ibrutinib therapy for chronic lymphocytic leukemia, France. A bright red, slightly painful lesion with a sharp border was localized on the left thigh.

Laboratory results revealed an abnormal leukocyte count of 13.5 G/L (reference range 4–10 G/L) and a neutrophil count of 10 G/L. We noted normal laboratory findings for C-reactive protein and gammaglobulin levels and found no evidence of progressive CLL. Skin biopsy showed eosinophilic spongiosis leading to spongiotic vesicles, perivascular/interstitial inflammatory infiltrates composed of eosinophilic and lymphocytic cells without atypical cells, mostly located in the superficial and mid dermis (Figure 2, panels A, B). Grocott methenamine silver stain, Gram stain, and periodic acid–Schiff stain showed no bacterial or fungal element. We performed 16S PCR testing directly on skin biopsy; results were negative. Two different aerobic blood culture bottles showed helical gram-negative rods after 96 hours of incubation; however, we could not identify the strain based on the blood culture broth. We obtained single colonies of fresh bacterial culture and performed matrix-assisted laser/desorption ionization time-of-flight (MALDI-TOF) mass spectrometry without success. We finally identified the strain as *Helicobacter cinaedi* by sequencing the entire 16S ribosomal RNA gene using 2 pairs of primers.

**Figure 2:**
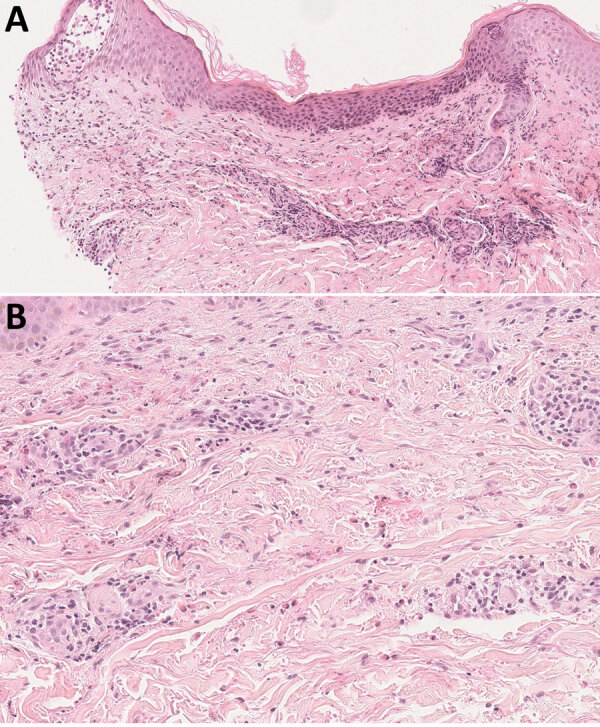
Histologic aspect of a skin lesion revealing *Helicobacter cinaedi* bacteremia in a man on ibrutinib therapy for chronic lymphocytic leukemia, France. Skin biopsy obtained from erythema on the right side of the abdomen (original magnification ×50 in panel A, ×100 in panel B) show eosinophilic spongiosis leading to spongiotic vesicles and inflammatory perivascular and interstitial infiltrate, mostly located in the superficial and mid dermis and composed of eosinophils and lymphocytes, with no atypical cells. Hematoxylin-eosin-saffron stains.

We treated the patient with amoxicillin/clavulanate for 6 weeks and discontinued ibrutinib. During the patient’s antibiotic therapy, we performed 3 consecutive blood cultures on 3 different days, all of which remained negative, confirming the efficiency of our antibiotic strategy. The patient achieved complete clinical remission without relapse 6 months after antibiotic discontinuation and was able to restart ibrutinib therapy at that time.

*Helicobacter* is a gram-negative spiral bacillus belonging to the Helicobacteriaceae family (*1*,*2*). It has been considered an opportunistic infection in patients with either acquired or primary immunodeficiencies (*3*,*4*). Cases of *H. cinaedi* bacteremia have been reported very rarely in immunocompetent persons (*5*). Reported clinical manifestations of *H. cinaedi* infection have included fever, proctitis, enteritis, cellulitis, and arthritis (*1*). Cutaneous manifestations are encountered mainly in the setting of *H. cinaedi* bacteremia, typically presenting as mild but painful cellulitis of the extremities (*6*).

*H. cinaedi* is a slow-growing bacterium and is difficult to identify. Traditional bacterial identification systems bear numerous limitations. As demonstrated in our report, a molecular approach is helpful in identifying the strain. Although MALDI-TOF mass spectrometry was insufficient to establish a diagnosis for this patient, others have used it successfully (*7*). A more efficient strategy would be combining a prolonged blood culture incubation time with MALDI-TOF mass spectrometry and a molecular approach. *H. cinaedi* is usually susceptible to carbapenems, tetracyclines, and aminoglycosides (*1*,*8*), as well as to amoxicillin and ceftriaxone (with intermediate MICs) (*1*,*6*). However, this species is resistant to macrolides, such as erythromycin and clarithromycin, and to ciprofloxacin (*1*,*8*). To avoid recurrence, prolonged therapies (2–8 weeks) are preferable (*6*).

*H. cinaedi* pathogenicity has not been clearly identified. The bacterium’s cytolethal distending toxin is reported as a potential virulence factor (*9*). Compared with other *Helicobacter* species, *H. cinaedi* might have a stronger ability to translocate from the intestinal tract to the vascular system, resulting in a greater chance for bacteremia (*1*). *H. cinaedi* is a known cause of bacteremia in patients with humoral immunodeficiency, especially X-linked agammaglobulinemia (*4*).

For our patient, given that CLL was quiescent and gammaglobulins levels were normal, ibrutinib may have promoted the occurrence of *H. cinaedi* bacteremia. Infections are common adverse events in patients receiving ibrutinib, especially pneumonia and invasive fungal infections (*10*). Because BTK inhibition in patients treated with ibrutinib does not generally result in immunoglobulin depletion (*10*), the increased risk for infection might be explained by other putative mechanisms associated with BTK. For example, ibrutinib’s disruption of chemokine-controlled B cell migration, trafficking, and homing to lymphoid organs might impair the humoral response to *H. cinaedi* (*10*). Furthermore, ibrutinib’s off-target inhibition of interleukin 2–inducible T-cell kinase could weaken the adaptive response against *H. cinaedi* (*10*). In addition, BTK is involved in Toll-like receptor signaling and is found in other immune cell subsets, such as neutrophils and macrophages. Thus, BTK inhibition could lead to a defect in host innate immune response or proliferation and function of myeloid cells.

The case we report illustrates 2 problems associated with *H. cinaedi* infections. First, patients must often endure prolonged courses of antibiotic therapy to avoid recurrence. Second, bacterial strains grow very slowly and often demand a molecular approach to establish microbiological diagnosis. Clinicians should add *H. cinaedi* infection to the diagnostic list when treating recurrent cellulitis, especially in patients with humoral immunodeficiency and those treated with ibrutinib.
